# Changes in Anxiety and Depression Traits Induced by Energy Restriction: Predictive Value of the Baseline Status

**DOI:** 10.3390/nu11061206

**Published:** 2019-05-28

**Authors:** Claudia Rodriguez-Lozada, Marta Cuervo, Amanda Cuevas-Sierra, Leticia Goni, Jose Ignacio Riezu-Boj, Santiago Navas-Carretero, Fermin Ignacio Milagro, Jose Alfredo Martinez

**Affiliations:** 1 Department of Nutrition, Food Science and Physiology, Center for Nutrition Research, University of Navarra, 31008 Pamplona, Spain; crodriguez.55@alumni.unav.es (C.R.-L.); acuevas.1@alumni.unav.es (A.C.-S.); lgoni@unav.es (L.G.); jiriezu@unav.es (J.I.R.-B.); snavas@unav.es (S.N.-C.); fmilagro@unav.es (F.I.M.); jalfmtz@unav.es (J.A.M.); 2 Navarra Institute for Health Research (IdiSNA), 31008 Pamplona, Spain; 3 CIBERobn, Fisiopatología de la Obesidad y la Nutrición; Carlos III Health Institute, 28029 Madrid, Spain

**Keywords:** overweight, weight loss, hypocaloric diet, macronutrient distribution, anxiety, depression

## Abstract

Current evidence proposes diet quality as a modifiable risk factor for mental or emotional impairments. However, additional studies are required to investigate the effect of dietary patterns and weight loss on improving psychological symptoms. The aim of this investigation was to evaluate the effect of energy-restriction, prescribed to overweight and obese participants, on anxiety and depression symptoms, as well as the potential predictive value of some baseline psychological features on weight loss. Overweight and obese participants (n = 305) were randomly assigned for 16 weeks to two hypocaloric diets with different macronutrient distribution: a moderately high-protein (MHP) diet and a low-fat (LF) diet. Anthropometrical, clinical, psychological, and lifestyle characteristics were assessed at baseline and at the end of the intervention. The nutritional intervention evidenced that weight loss has a beneficial effect on trait anxiety score in women (β = 0.24, *p* = 0.03), depression score in all population (β = 0.15, *p* = 0.02), particularly in women (β = 0.22, *p* = 0.03) and in subjects who followed the LF diet (β = 0.22, *p* = 0.04). Moreover, weight loss could be predicted by anxiety status at baseline, mainly in women and in those who were prescribed a LF diet. This trial suggests that weight loss triggers an improvement in psychological traits, and that anxiety symptoms could predict those volunteers that benefit most from a balanced calorie-restricted intervention, which will contribute to individualized precision nutrition.

## 1. Introduction

Obesity is a growing public health problem worldwide [1], which is an important risk factor for inducing certain chronic diseases [2]. In this context, it has been reported that there is a negative relationship between obesity and life satisfaction, due to the emotional cost of body image dissatisfaction, including low self-esteem, depression, and other adverse emotional states [3]. Anxiety and depression disorders are among the most common mental impairments in the community and in primary care settings [4]. Patients with at least one of those disorders are more likely to report physical complaints rather than mental health manifestations, whose symptoms may initially seem vague and non-specific [4]. Besides obesity, anxiety and depression have been related to other chronic illnesses such as diabetes [5], rheumatic diseases [6], cardiovascular diseases [7], or chronic obstructive pulmonary disease [8].

Cognitive behavioral therapy and pharmacotherapy have been two emerging theoretical approaches for psychological management [9]. However, the population burden of these disorders has not been reduced, despite an increase in the application of both treatment strategies [10].

In this context, anxiety is a normal and understandable reaction that provides an alerting signal, and improves physical and mental performance [11]. When anxiety is excessive or occurs without provocation, it may be said to be abnormal or pathological, which results in a deterioration in general performance and in emotional and physical discomfort [12]. Furthermore, depressive disorders are characterized by disturbed sleep and appetite, as well as feelings of guilt or low self-worth, poor concentration, and even medically unexplained symptoms [13]. An individual with a mild depressive episode will have some difficulty in continuing with ordinary work and social activities, while during a severe depressive episode, it may substantially impair an individual’s ability to cope with daily life [13].

Current evidence from systematic reviews has proposed diet quality as a modifiable risk factor for mental illnesses [14]. Observational studies have reported the association between dietary intake and emotional features. Nevertheless, additional intervention studies are required to investigate the effect of dietary patterns, specific nutrients or micronutrient combinations, as well as weight loss on improving or controlling psychological symptoms [14]. Obesity has been associated with physiological impairments, where a reduction in weight focused on caloric restriction with a good adherence, and an increase in physical activity may produce overall health benefits, including psychological traits [15]. Indeed, obesity is not only influenced by energy intake and energy expenditure, but also by psychological, cultural, genetic, and social features [16,17]. These characteristics should be assessed at the beginning of a weight loss intervention to predict the individualized response of each subject. Therefore, the main objective of the present trial was to study the effect of two hypocaloric diets with different macronutrient distribution on anxiety and depression symptoms, and the putative baseline predictive value of certain psychological symptoms on future weight loss.

## 2. Methods

### 2.1. Participants

**Figure 1:**
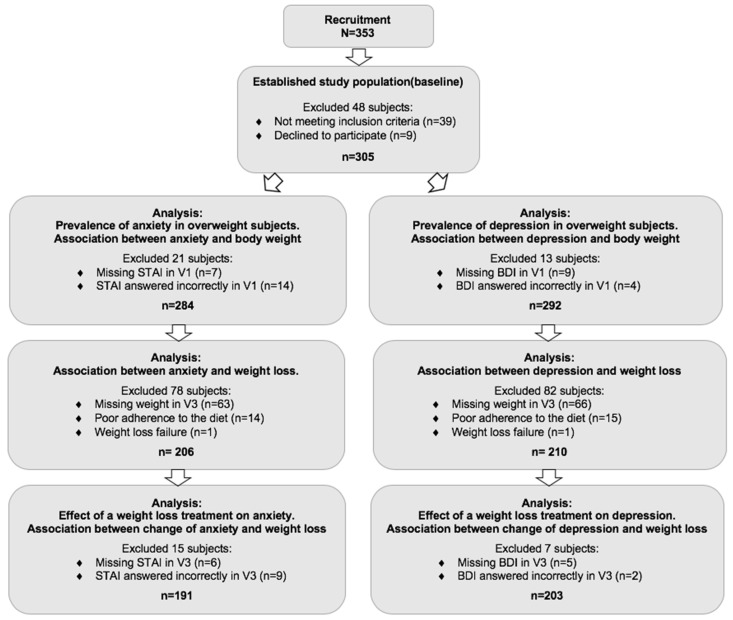
Flow chart of study population. Abbreviations: STAI = State-Trait Anxiety Inventory; BDI = Beck Depression Inventory.

This longitudinal study enrolled Spanish adults of self-reported European ancestry, aged 18–70 years, who met the inclusion criteria established by the program, a body mass index (BMI) between 25 and 40 kg/m^2^ and a physical examination, which was considered normal or clinically insignificant by the researcher. Volunteers were recruited at the Center for Nutrition Research of the University of Navarra in the city of Pamplona, Navarra, Spain. Major exclusion criteria included subjects reporting weight change greater than 3 kg within the 3 months before the study; pregnant or lactating women; a clinical history of cardiovascular disease, type 1 or type 2 diabetes treated with insulin; and unstable medication dose for hyperlipidemia and/or hypertension. Subjects taking weight loss medication or other drugs that affect body weight were also excluded. A total of 305 subjects, men and women, met the criteria to be part of this study, some were discarded due to missing data for the purpose of the study (Figure 1). This investigation followed the ethical principles for medical research in humans from the 2013 Helsinki Declaration [18]. All participants provided written informed consent after they received an information sheet and additional verbal explanation. The study protocol was approved by the Research Ethics Committee of the University of Navarra (ref. 132/2015) and was registered at Clinical Trials (reg. no. NCT02737267).

### 2.2. Study Design

**Figure 2:**
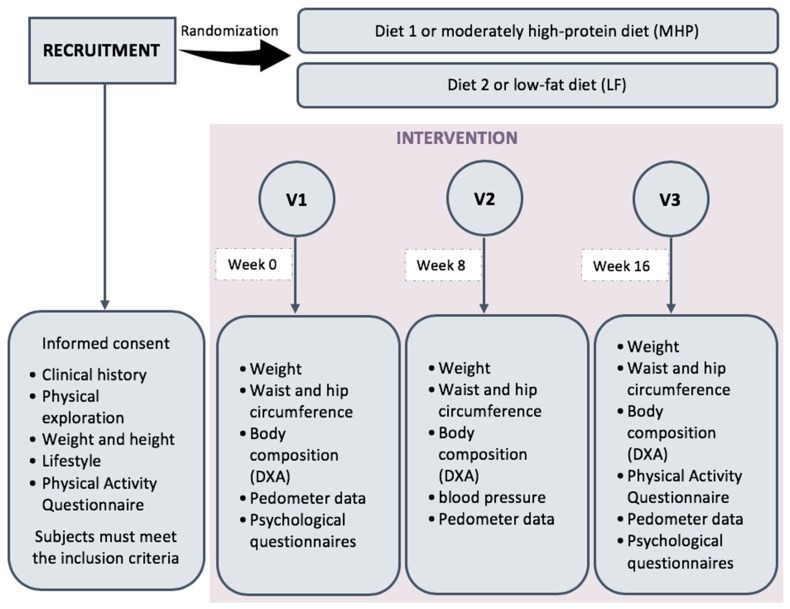
General schedule of the study.

The present survey was a randomized, longitudinal, and controlled intervention trial to evaluate the response to two energy-restricted diets with varied macronutrient distribution. Diet 1 was moderately high in protein or MHP (30% of calorie intake provided by proteins, 30% by fat, and 40% provided by carbohydrates), whereas diet 2 was low in fat or LF (18% of calorie intake provided by proteins, 22% by fat and 60% provided by carbohydrates). In both cases, a 30% calorie restriction was prescribed regarding the individual’s energy expenditure in order to create a negative energy balance that stimulates weight loss. The intervention period lasted a total of 16 weeks (Figure 2). The adherence to the diets was graded by trained dietitians during each visit of the intervention according to the following scale: 0: failure to follow the diet at any time (poor adherence); 1: follow-up across weekdays but not during weekends (regular adherence); 2: occasionally exceeded from recommendations (good adherence); and 3: continuous follow-up (very good adherence). For the purpose of this study, the mean adherence punctuation between visit 2 and visit 3 was used. Participants had four scheduled visits at the Nutrition Research Centre of the University of Navarra (Figure 2).

### 2.3. Anthropometry and Physical Activity

Anthropometric measurements such as weight, height, and waist circumference (WC) were measured in fasting conditions by a trained dietitian, as previously described [19]. Body mass index (BMI) was calculated as the ratio between weight and squared height (kg/m^2^). Total body fat (TFAT, %) and visceral fat (VFAT, Kg) contents were determined by dual-energy x-ray absorptiometry as described by the supplier (Lunar Prodigy, software version 6.0, Madison, WI, USA). Physical activity level was assessed through a validated questionnaire [20], keeping in mind that volunteers had to maintain their usual physical activity habits during the study. The volume of activity was expressed in metabolic equivalents (METs), as described elsewhere [21].

### 2.4. Anxiety and Depression Symptoms

Anxiety was assessed using the validated Spanish translation of the State-Trait Anxiety Inventory (STAI). The purpose of the STAI is to measure the presence and severity of current symptoms of anxiety and a generalized propensity to be anxious via self-report [15]. The questionnaire contains 40 items and has two subscales within this measure. First, the State Anxiety Scale (S-Anxiety) evaluates the current state of anxiety, asking how respondents feel “right now,” ranging from 0 to 60. Second, the Trait Anxiety Scale (T-Anxiety) evaluates relatively stable aspects of “anxiety proneness”. Higher values of the final score indicate greater state-trait anxiety symptoms, ranging from 0 to 60 [22].

Concerning depression examination, the validated Spanish translation of the Beck Depression Inventory (BDI) was used, the purpose being to measure depression symptoms and the severity in participants aged ≥13 years [23]. This questionnaire contains 21 items, each with four possible responses. Each response is assigned a score ranging from 0 to 3, indicating the severity of the symptom. Items 1 to 13 assess symptoms that are psychological in nature, while items 14 to 21 assess more physical symptoms [24]. Question 19 is related to an anorexigenic symptom, thus, when it comes to people who have been on a diet treatment it is recommended that the value of that alternative is discarded [25]. For that reason, question 19 was considered at the beginning of the trial, but not at the end of the intervention, given that volunteers were already following a hypocaloric diet.

### 2.5. Statistical Analyses

In order to detect a minimum difference of weight loss of approximately 2 kg ± 5 kg between dietary groups, the required sample size was estimated at 200 individuals (α = 0.05 and statistical power of 80%). However, considering a potential dropout rate of 30%, we considered recruiting more than 260 subjects. Descriptive characteristics were presented using means and 95% confidence intervals for continuous variables. Differences at baseline between men and women, as well as between MHP and LF dietary groups were assessed using unpaired Student *t*-test. For the analysis of significant differences in anthropometrical and psychological outcomes between visit 1 and visit 3, a paired Student t-test was used. An ANCOVA test adjusted for sex, age, diet, and smoking habit was carried out for comparing changes between men and changes between women, as well as changes between volunteers in the MHP diet group and changes between volunteers in the LF diet group. Confounder variables were categorized as follows: sex (male/female), diet (MHP/LF), and smoking (smoker/former smoker/non-smoker). Age was considered as a continuous variable. Physical activity level and energy intake at baseline were also considered as cofactors in the initial data analysis, however, no statistically significant differences were observed after considering them. Linear regression analyses were carried out to evaluate predictive relationships between psychological symptoms at baseline and weight loss, and between weight loss and changes in psychological features. The model was adjusted for age, sex, and smoking habit. Statistical analyses were carried out using STATA (Statistical Software for Professional for StataCorp, College Station, TX, USA) version 12. The statistical significance was set at *p* < 0.05.

## 3. Results

Anthropometric, clinical, psychological, and lifestyle characteristics of the volunteers, classified by sex and diet, are presented (Table 1). As expected, there were differences between men and women regarding weight, WC, TFAT, VFAT, and physical activity at baseline. The prevalence of psychological features showed that the score of depression in women was significantly higher than in men (7.3 versus 4.9, *p* < 0.001). However, the opposite tendency was found for S-anxiety (*p* = 0.07). The prevalence of T-anxiety was not significantly different among men and women in this population (*p* = 0.10) at the beginning of the study. Moreover, between MHP diet and LF diet groups, there were no significant differences in anthropometric, clinical or lifestyle measurements at baseline (Table 1).

After 16 weeks of the dietary intervention, there were significant changes in most analyzed anthropometric and psychological variables. Weight, BMI, WC, TFAT, and VFAT showed a significant decrease in the whole population during the intervention, both in men and women, and after following the MHP diet or the LF diet. The only variable that did not vary throughout the intervention was physical activity. According to psychological features, there was a significant improvement on depression symptoms in all participants, which remained significant in all study groups when sorted by sex and diet group. This improvement was significantly higher in women (Table 2). Regarding the changes in T-anxiety scores, the improvement in all participants only remained significant among women and participants who consumed the MHP diet. No relevant changes were observed regarding S-anxiety scores at the end of the intervention period, not in total population, nor when sorted by sex and type of diet. All these results obtained by a “per protocol” model were maintained when the “intention to treat” model was applied. Data concerning T-anxiety evidenced a statistically marginal significance (*p* = 0.10), which was significant under one-tail criteria.

**Figure 3:**
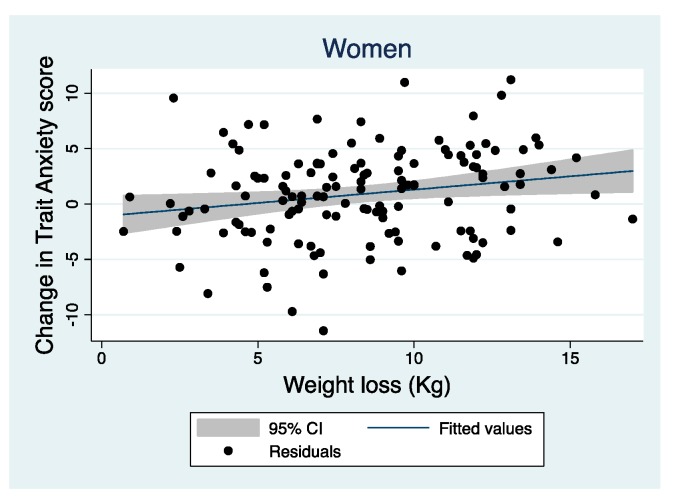
Relationship between weight loss and change in Trait Anxiety score in women, adjusted by age, diet and smoking habit.

**Figure 4:**
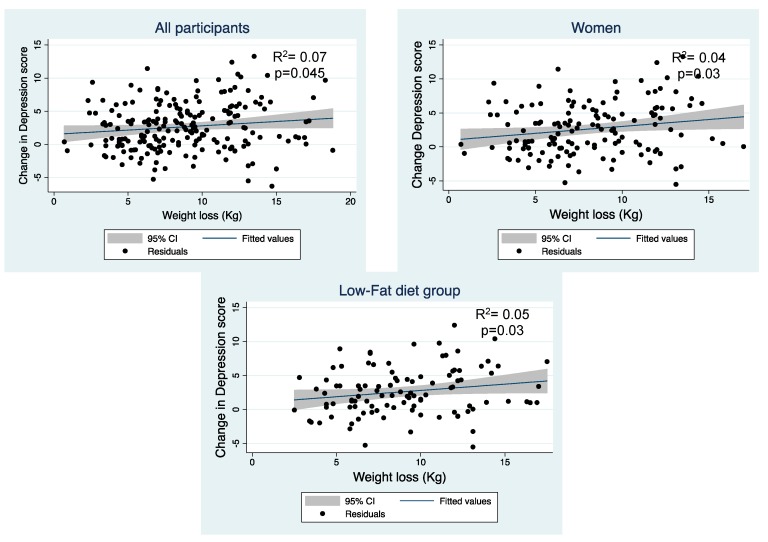
Relationship between weight loss and change in depression score in all participants, women and Low-Fat diet group, adjusted by age, diet, and smoking habit.

To study if the changes observed in depression and T-anxiety symptoms throughout the intervention were related to weight loss, the magnitude of the associations were studied. According to T-anxiety, a significant association between weight loss and the improvement of those symptoms in women, but not in men were observed (Table 3 and Figure 3). However, changes in the depression score in all volunteers were significantly associated with weight loss, especially in women and in the LF diet group (Table 3 and Figure 4). No significant relationship was found regarding changes in S-anxiety score.

**Figure 5:**
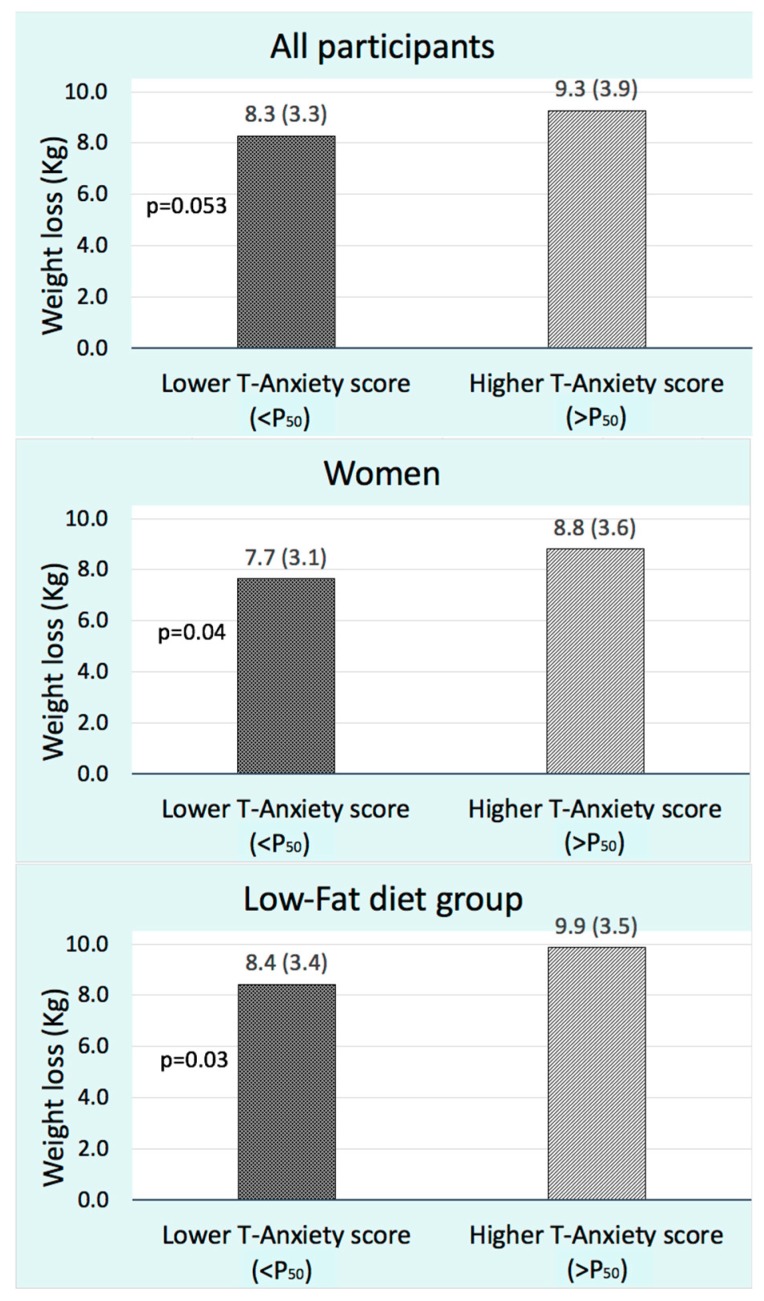
Mean weight loss in participants with lower and higher Trait Anxiety score at baseline. The *p*-value was calculated by unpaired Student’s *t*-test.

No relevant association was found between weight loss and S-anxiety scores at the beginning of the intervention (Table 4). However, in all participants, T-anxiety symptoms at baseline were significantly associated with weight loss. This predictive association remained significant in women and in the LF diet group after classifying the study population by sex and type of diet, respectively. Therefore, these groups were categorized for the T-anxiety score according the median value or 50th percentile (P_50_). The result was that those with a higher T-anxiety score at baseline presented a greater weight loss (Figure 5). Regarding, depression symptoms, a marginal trend towards significance was found in those volunteers that received a LF diet (β = 0.42, *p* = 0.06).

## 4. Discussion

The aim of this study was to investigate the effect of weight loss induced by two calorie-restricted diets on anxiety and depression symptoms, and to analyze the role of the baseline psychological features in predicting an eventual subsequent weight loss.

After weight loss induced by following a hypocaloric diet, a significant change in anthropometric and body composition outcomes was observed, as described by others [26,27], leading to a lower risk for metabolic disturbances and other benefits [28,29], including the improvement of self-esteem [30].

In the current trial, significantly higher scores of depression symptoms were found in women at baseline. In accordance with our results, a number of authors [12,31] have reported that women tend to have higher levels of anxiety and depression than men. Psychological characteristics were not significantly different at baseline between the MHP diet and the LF diet group as expected, given that the study population was randomly categorized into two parallel balanced groups.

With regard to the effect of a weight loss intervention, this study showed that depressive symptoms improved significantly in all the volunteers of the study. This beneficial outcome was stronger in women, with a decrease in BDI score of 43%, while in men it was about 31%. Previous studies reported an improvement in depressive symptoms during a weight loss treatment both, in hospitalized patients [32,33], and in healthy population [34,35,36]. This fact could be influenced by the adoption of exercise [33]. However, in this study there was no variation in physical activity given and it was prescribed to the volunteers to maintain their usual physical activity habits during the intervention. Another factor that could have an important role is the macronutrient composition of the weight-loss diet. Both diets remained within the recommendations for adults proposed by the U.S. Institute of Medicine, and our previous studies [37,38,39]. Nowadays, there is consistent epidemiological evidence about an inverse relationship between a healthy dietary pattern, characterized by a high intake of fruits, vegetables, whole-grains, olive oil, fish, and a lower risk of depression [40,41]. On the other hand, unhealthy diets have been proven to be associated with depression and anxiety [42]. In the present study, participants that lost more weight showed a larger amelioration in depression symptoms, as previously reported in another study that included a cognitive-behavioral weight loss intervention [43]. Similarly, there is evidence that emotional features as perception of body weight/shape could be an important mediator of the association between obesity and physical or mental health impairments not only in women but also in men [44,45,46]. In such trial, the intervention was mainly nutritional, therefore, the addition of more strategies like physical activity and psychological sessions, may favor a stronger association between weight loss and improvements of psychological features after the intervention.

When referring to T-anxiety, there was a significant improvement of the symptoms after the weight loss diet, which was more evident in women and in volunteers who received the MHP diet. With regard to women, a fully adjusted analysis showed that the amelioration in T-anxiety symptoms was significantly predicted by weight loss. In association with S-anxiety, no relevant changes were reported in the study population. In fact, the score in both men and women and in participants who received a MHP and LF diet showed a tendency to increase at the end of the intervention. Given that this scale evaluates a momentary condition, where the intensity of unpleasant feelings depends on the situation, that increasing trend may be due to fear of regaining weight after the intervention period, as proposed in a previous study [47].

Our research suggests that greater T-anxiety symptoms at the beginning of the intervention could predict a higher weight loss. After stratification by sex and diet, this association remained significant among women and participants who received the LF diet. These results could be explained by the fact that perfectionism could act as a cause and symptom of generalized anxiety disorder, therefore, it is more likely that anxious people follow closely the dietary recommendations [48]. Our results are consistent with a previous investigation that found a significant correlation between the neuroticism personality trait and successful weight loss [49]. In such intervention, anxiety was considered as one of the facets of neuroticism [50].

The presence of depression symptoms at baseline was not found to have a significant predictive effect on weight loss in this population, as previously reported in an intervention with obese patients following a conventional weight loss treatment [51]. Most studies that have reported positive associations were carried out with subgroups with more vulnerable individuals, such as patients undergoing obesity surgery [52,53]. Either way, the findings of the present study do not agree with the general belief that the most anxious or depressive people respond worse to a weight loss treatment, but it should be considered that we evaluated stages in depression/anxiety manifestations. Nevertheless, it is recommended to establish the difference between psychological disorders, which require clinical assessment, and psychological symptoms. In any case, psychological conditions are closely related not only with mental diseases, but also to systemic disorders such as obesity, diabetes, and cardiovascular diseases, among others [5,6,7,8] and their assessment in the medical treatments is important.

Both STAI and BDI are the most widely used scale tools in research worldwide to evaluate anxiety and depression symptoms [33]. Although it is a limitation of the study that these psychological tests are self-administered, they are validated. Moreover, they made it possible for this study to evaluate the associations between mild psychological distress and weight loss. Another important limitation is that there was no assessment of body dissatisfaction (BD) or eating-disordered behavior. Evidence suggests that BD is a potent mediator of the association between obesity and impairment in psycho-social functioning in both men and women [44,45]. Furthermore, and while BD remains more common in women than in men [54], its adverse impact on psychological functioning and strong association with eating disorder behaviors, such as binge eating, extreme dietary restriction and extreme weight-control behaviors, does not appear to differ by sex [45,55]. Although the statistical analysis includes covariates, such as sex, age and smoking, it would have been interesting to measure BD or eating disorders and include them in the data analysis, given the role that they play in mediating the association between weight loss and improvement in psycho-social functioning, particularly in women [46]. An important strength of this study was the fact that anthropometric and body composition measurements were taken by an experienced dietitian unique for each patient, which made the data more reliable than if they had been self-reported. Moreover, they were in charge of monitoring the volunteers while filling in the psychological tests in order to ensure that the questionnaires collected were answered properly and that the amount of missing data was minimal. Finally, for this intervention the evaluation of T-anxiety was included, which is a more stable construct than S-anxiety. Although T-anxiety evaluation is not used very often in clinical studies, especially in those aimed to relate this symptom to obesity or its relationship with a weight loss treatment. This scale has been previously used to study the association between T-anxiety with metabolic syndrome [53] and also in surgical patients, where it was a more reliable predictor than S-anxiety when it came to physiological measures [56]. Bearing in mind that S-anxiety measures a transitory emotional state and easily varies according to the situation, T-anxiety could be considered a more useful scale when it comes to measure the effect of a weight-loss intervention on anxiety symptoms. In the future, it would be interesting to measure BD as well as to include participants with higher levels of anxiety and/or depression for a more accurate measure of the variation in the psychological response to a weight loss treatment. It would also be helpful to follow participants after the intervention period to assess if they maintain their weight loss and its relationship with psychological symptoms.

## 5. Conclusion

The current investigation indicates that weight loss through interventions with hypocaloric diets could improve depression symptoms in the whole study population, with a particularly prevalent effect in women. Likewise, weight loss improved trait-anxiety symptoms in the analyzed sample, specifically in women and in volunteers who were prescribed the MHP diet. In addition, greater symptoms of T-anxiety at the beginning of a hypocaloric diet intervention could predict a higher weight loss, especially in women and in participants who were fed a LF diet. These results, in addition to the positive effect on body composition, show that weight loss could translate into an integral improvement of health within precision nutrition approaches.

## Figures and Tables

**Table 1:** Anthropometric, clinical, psychological, and lifestyle baseline characteristics of participants categorized by sex and type of diet.

		Sex		Diet	
	All Population *n* = 305 mean (95%CI)	Men *n* = 92 mean (95%CI)	Women *n* = 213 mean (95%CI)	MHP *n* = 146 mean (95%CI)	LF *n* = 159 mean (95%CI)
**Women***n* (%)	213 (71.0)	-	-	101 (47.4)	112 (52.6) ^1^
**Obese***n* (%)	190 (62.3)	62 (67.4)	128 (60.1)	90 (61.6)	100 (62.9)
**Smokers***n* (%)	66 (21.8)	27 (29.4) *	39 (18.4)	27 (18.8)	39 (24.5)
**Age (years)**	45.3 (44.1; 46.5)	45.7 (43.6; 47.7)	45.2 (43.7; 46.6)	44.8 (43.1; 46.6)	45.8 (44.2; 47.4)
**Weight (Kg)**	87.7 (86.2; 89.2)	97.4 (94.9; 99.8) **	83.5 (81.9; 84.9)	87.6 (85.4; 89.7)	87.8 (85.8; 89.8)
**BMI (Kg/m^2^)**	31.6 (31.2; 32.0)	31.7 (31.1; 32.4)	31.6 (31.1; 32.1)	31.4 (30.9; 31.9)	31.8 (31.2; 32.4)
**Waist circumference (cm)**	102.2 (100.9; 103.4)	108.3 (106.5; 110.2) **	99.6 (98.2; 100.9)	102.5 (100.8; 104.3)	101.9 (100.2; 103.5)
**Total fat mass (%)**	36.9 (36.0; 37.8)	34.7 (33.2; 36.3) *	37.8 (36.8; 38.8)	37.0 (35.8; 38.2)	36.8 (35.5; 38.1)
**Visceral fat mass (Kg)**	1.48 (1.37; 1.58)	2.31 (2.12; 2.50) **	1.11 (1.03; 1.19)	1.48 (1.33; 1.63)	1.47 (1.33; 1.61)
**Physical activity (METs)**	23.8 (21.5; 26.0)	31.2 (26.2; 36.2) **	20.5 (18.2; 22.8)	22.0 (18.9; 25.2)	25.2 (21.9; 28.5)
**State Anxiety (points)**	24.7 (24.2; 25.2)	25.4 (24.5; 26.2)	24.4 (23.8; 24.9)	24.9 (24.2; 25.7)	24.5 (23.8; 25.1)
**Trait Anxiety (points)**	25.5 (24.9; 26.1)	24.9 (23.9; 25.8)	25.8 (25.2; 26.5)	25.8 (24.9; 26.6)	25.3 (24.7; 26.0)
**Depression (points)**	6.6 (6.0; 7.1)	4.9 (4.1; 5.8) **	7.3 (6.6; 7.9)	6.7 (5.9; 7.5)	6.4 (5.7; 7.1)

Abbreviations: MHP = moderately high-protein; LF = low fat; BMI = body max index. * *p* ≤ 0.05, ** *p* ≤ 0.001. Comparisons between sex or diets by unpaired Student *t*-test and Chi-squared test for continuous and categorical variables. ^1^ Comparison between men and women included in MHP and LF diets (*p* = 0.783).

**Table 2:** Changes in anthropometric and psychological outcomes after the 16-weeks energy-restricted intervention in men and women, as well as depending on the prescribed diet.

	All Population *n* = 217	Men *n* = 66	Women *n* = 151	MHP Diet *n* = 104	LF *n* = 113	*p ^a^*	*p ^b^*
	Mean (95%CI)	Mean (95%CI)	Mean (95%CI)	Mean (95%CI)	Mean (95%CI)		
**Δ Weight (Kg)**	−8.6 (−8.1; −9.1) **	−10.0 (−9.0; −11.0) **	−8.0 (−7.5; −8.6) **	−8.3 (−7.6; −9.1) **	−8.9 (−8.3; −9.6) **	*	ns
**Δ BMI (Kg/m^2^)**	−3.1 (−2.9; −3.3) **	−3.3 (−2.9; −3.6) **	−3.1 (−2.9; −3.3) **	−3.0 (−2.7; −3.3) **	−3.2 (−2.9; −3.5) **	ns	ns
**Δ WC (cm)**	−9.1 (−8.5; −9.7) **	−10.6 (−9.6; −11.6) **	−8.5 (−7.8; −9.2) **	−8.8 (−7.9; −9.7) **	−9.4 (−8.7; −10.2) **	*	ns
**Δ TFAT (%)**	−6.9 (−6.5; −7.3) **	−8.3 (−7.5; −9.1) **	−6.3 (−5.8; −6.8) **	−6.8 (−6.1; −7.4) **	−7.0 (−6.5; −7.6) **	**	ns
**Δ VFAT (Kg)**	−0.5 (−0.4; −0.5) **	−0.9 (−0.8; −1.0) **	−0.3 (−0.3; −0.3) **	−0.5 (−0.4; −0.6) **	−0.5 (−0.4; −0.6) **	**	ns
**Δ PA (METs)**	−1.1 (1.6; −3.7)	−1.6 (4.1; −7.3)	−0.9 (2.0; −3.7)	0.7 (4.7; −3.3)	−2.7 (0,7; −6.2)	ns	ns
**Δ State Anxiety (points)**	0.2 (0.8; −0.5)	0.4 (1.5; −0.7)	0.1 (0.9; −0.7)	0.1 (1.0; −0.9)	0.3 (1.1; −0.5)	ns	ns
**Δ Trait Anxiety (points)**	−0.7 (−0.1; −1.3) *	−0.2 (0.9; −1.3)	−0.9 (−0.2; −1.7) *	−1.1 (−0.2; −2.0) *	−0.3 (0.5; −1.2)	ns	ns
**Δ Depression (points)**	−2.7 (−2.1; −3.2) **	−1.5 (−0.7; −2.4) **	−3.1 (-2.5; -3.8) **	−2.8 (−2.0; −3.6) **	−2.5 (−1.8; −3.2) **	*	ns

Abbreviations: Δ = change; MHP = moderately high-protein; LF = low fat; BMI = body max index; TFAT = total fat mass; VFAT = visceral fat mass; and PA = physical activity. Note, asterisks within columns (all population, sex, and diets) refers to the difference between the baseline and the end. ^a^ Comparison between changes men and women adjusted for diet, age, and smoking. ^b^ Comparison between changes in MHP diet group and LF diet group adjusted for sex, age, and smoking. * *p* ≤ 0.05, ** *p* ≤ 0.001. Comparison of means between sex or diets by paired Student *t*-test. Comparison of changes by ANCOVA test adjusted by sex, diet, age, and smoking habit.

**Table 3:** Effect of weight loss induced by a calorie-restricted diet on changes in psychological symptoms.

	Δ S-anxiety Score		Δ T-anxiety Score		Δ Depression Score	
	β (95% CI)	*p* Value	β (95% CI)	*p* Value	β (95% CI)	*p* value
All population ^a^	0.04 (−0.16; 0.23)	0.71	0.09 (−0.08; 0.27)	0.28	0.15 (0.004; 0.29) *	0.045
Men ^b^	0.07 (−0.24; 0.38)	0.64	−0.16 (−0.48; 0.16)	0.32	0.01 (−0.21; 0.23)	0.92
Women ^b^	0.03 (−0.22; 0.27)	0.81	0.24 (0.03; 0.46) *	0.03	0.22 (0.03; 0.41) *	0.03
MHP Diet ^c^	0.11 (−0.18; 0.41)	0.46	0.04 (−0.20; 0.29)	0.72	0.08 (−0.13; 0.29)	0.46
LF Diet ^c^	−0.05 (−0.31; 0.21)	0.69	0.15 (−0.11; 0.42)	0.25	0.22 (0.01; 0.43) *	0.04

Abbreviations: MHP = moderately high-protein; LF = low fat. ^a^ Adjusted for sex, age, diet and smoking habit. ^b^ Adjusted for age, diet and smoking habit. ^c^ Adjusted for sex, age and smoking habit. *p ≤ 0.05. The *p*-value was calculated by Linear Regression analyses.

**Table 4:** Effect of baseline psychological symptoms on weight loss.

	S-anxiety Score		T-anxiety Score		Depression Score	
	β (95% CI)	*p* VALUE	β (95% CI)	*p* Value	β (95% CI)	*p* Value
All population ^a^	0.07 (−0.05; 0.18)	0.26	0.11 (0.003; 0.21) *	0.04	0.08 (−0.03; 0.19)	0.14
Men ^b^	0.02 (−0.23; 0.26)	0.89	0.05 (−0.16; 0.27)	0.64	0.11 (−0.16; 0.37)	0.41
Women ^b^	0.08 (−0.05; 0.21)	0.24	0.15 (0.03; 0.28) *	0.02	0.08 (−0.04; 0.19)	0.18
MHP Diet ^c^	0.03 (−0.14; 0.19)	0.76	0.02 (−0.14; 0.19)	0.81	0.02 (−0.13; 0.18)	0.77
LF Diet ^c^	0.09 (−0.06; 0.26)	0.23	0.20 (0.06; 0.35) **	0.006	0.14 (−0.01; 0.29)	0.06

Abbreviations: MHP = moderately high-protein; LF = low fat. ^a^ Adjusted for sex, age, diet and smoking habit. ^b^ Adjusted for age, diet and smoking habit. ^c^ Adjusted for sex, age and smoking habit. * *p* ≤ 0.05, ** *p* ≤ 0.01. The *p*-value was calculated by Linear Regression analyses.
